# Correlation of polygenic risk score and clinical phenotype in patients with genetic generalized epilepsy

**DOI:** 10.1002/epi.70444

**Published:** 2026-08-22

**Authors:** Sophie von Brauchitsch, Nils Hartung, Robin A. Karge, Bobby Koeleman, Samuel F. Berkovic, Danielle M. Andrade, Francesca Bisulli, Antonio Gambardella, Renzo Guerrini, Ingrid E. Scheffer, Ingo Helbig, Wolfram S. Kunz, Holger Lerche, Hiltrud Muhle, Christian Boßelmann, Savvas S. Papacostas, Mark I. Rees, Sanjay M. Sisodiya, Pasquale Striano, Lynnette Sadleir, Yvonne Weber, Dennis Lal, Costin Leu, Philipp S. Reif, Stefan Wolking, Felix Rosenow, Karl Martin Klein, for the Epi25 Collaborative

**Affiliations:** ^1^ Epilepsy Center Frankfurt Rhine‐Main, Department of Neurology University Medical Center and Goethe University Frankfurt Frankfurt Germany; ^2^ Center for Personalized Translational Epilepsy Research Goethe University Frankfurt Frankfurt Germany; ^3^ Section of Epileptology, Department of Neurology RWTH Aachen University Aachen Germany; ^4^ Department of Medical Genetics University Medical Center Utrecht, Utrecht University Utrecht the Netherlands; ^5^ Epilepsy Research Centre University of Melbourne, Austin Health Melbourne Victoria Australia; ^6^ Adult Genetic Epilepsy Program, Division of Neurology Krembil Brain Institute, University of Toronto Toronto Ontario Canada; ^7^ IRCCS Istituto delle Scienze Neurologiche di Bologna, full member of the European Reference Network EpiCARE Bologna Italy; ^8^ Department of Biomedical and Neuromotor Sciences University of Bologna Bologna Italy; ^9^ Department of Medical and Surgical Sciences Magna Græcia University of Catanzaro Catanzaro Italy; ^10^ Pediatric Neurology Unit, A. Meyer Children's Hospital University of Florence Florence Italy; ^11^ Department of Paediatrics University of Melbourne Melbourne Victoria Australia; ^12^ Murdoch Children's Research Institute Parkville Victoria Australia; ^13^ Florey Institute of Neuroscience and Mental Health Melbourne Victoria Australia; ^14^ Department of Neuropediatrics University Medical Center Schleswig‐Holstein, Christian Albrecht University of Kiel Kiel Germany; ^15^ Division of Neurology Children's Hospital of Philadelphia Philadelphia Pennsylvania USA; ^16^ Epilepsy NeuroGenetics Initiative Children's Hospital of Philadelphia Research Institute Philadelphia Pennsylvania USA; ^17^ Department of Epileptology University Bonn Medical Center, University of Bonn Bonn Germany; ^18^ Department of Neurology and Epileptology Hertie Institute for Clinical Brain Research, University Clinic Tübingen, Eberhard Karl University of Tübingen Tübingen Germany; ^19^ Neurophysiology Department Cyprus Institute of Neurology & Genetics Nicosia Cyprus; ^20^ Neurology Research Group, Institute of Life Science, Faculty of Medicine, Health, & Life Sciences Swansea University Swansea UK; ^21^ Research Department of Epilepsy, University College London Queen Square Institute of Neurology University College London London UK; ^22^ Chalfont Centre for Epilepsy Chalfont St Peter UK; ^23^ Department of Neuroscience, Rehabilitation, Ophthalmology, Genetics, and Mother‐Child‐Sciences University of Genoa Genoa Italy; ^24^ IRCCS Istituto Giannina Gaslini, full member of European Reference Network EPICARE Genoa Italy; ^25^ Department of Paediatrics and Child Health University of Otago Wellington New Zealand; ^26^ Genomic Medicine Institute Lerner Research Institute, Cleveland Clinic Cleveland Ohio USA; ^27^ Stanley Center for Psychiatric Research Broad Institute of Harvard and Massachusetts Institute of Technology Cambridge Massachusetts USA; ^28^ Cologne Center of Genomics University of Cologne Cologne Germany; ^29^ Department of Neurology, McGovern Medical School University of Texas Health Science Center at Houston Houston Texas USA; ^30^ Epilepsy Center Neurological Institute, Cleveland Clinic Cleveland Ohio USA; ^31^ Department of Clinical and Experimental Epilepsy Institute for Neurology, University College London London UK; ^32^ Department of Clinical Neurosciences, Medical Genetics, and Community Health Sciences, Hotchkiss Brain Institute & Alberta Children's Hospital Research Institute, Cumming School of Medicine University of Calgary Calgary Alberta Canada

**Keywords:** complex inheritance, EEG, epilepsy genetics, genotype–phenotype correlation, heritability, pharmacoresponse

## Abstract

**Objective:**

The polygenic risk score (PRS) for individuals with genetic generalized epilepsy (GGE) quantifies the common risk variants in genes identified in genome‐wide association studies. We hypothesized that the phenotype of GGE patients differs based on their GGE PRS.

**Methods:**

We identified participants with highest (*n* = 59) versus lowest (*n* = 48) PRS from the GGE patients (*n* = 2256) recruited through the Epi25 Collaborative for comparison. Detailed clinical data were acquired retrospectively for the 59 high PRS and 48 low PRS individuals with GGE from the Epi25 database and from the contributing centers. For validation, we accessed a larger cohort (*n* = 1175) of patients with GGE included in the Epi25 Collaborative.

**Results:**

This study found no difference in phenotypic features of patients between the high‐PRS GGE and low‐PRS GGE subgroups, including age at onset, family history, and specific GGE syndrome. However, more patients from the lowest compared to the highest PRS subgroup were pharmacoresistant (31.7% vs. 8.9%, *p* = .01). On validation in a larger cohort, the PRS did not differ in the group of pharmacoresistant compared to nonpharmacoresistant patients.

**Significance:**

No meaningful association between PRS and age at onset, history of febrile seizures, pre‐/perinatal complications, epilepsy syndromes, seizure types, co‐occurrence of functional/dissociative (nonepileptic) seizures, psychiatric comorbidities, electroencephalographic/magnetic resonance imaging findings, or drug response could be demonstrated in this study of people with GGE.

**Key points**

- We compared phenotypes of individuals with genetic generalized epilepsy and highest versus lowest polygenic risk scores.
- No difference in phenotypic features including age at onset, epilepsy syndrome, and family history was detected.
- A higher rate of pharmacoresistance was initially detected in the lowest polygenic risk score subgroup, but this finding was not confirmed in a larger cohort.
- No meaningful association between polygenic risk score and clinical phenotypic features or drug response could be demonstrated for GGE patients.

## 1 INTRODUCTION

Genetic generalized epilepsies (GGEs) represent 15%–20% of all epilepsies.^1^ GGE includes patients with idiopathic generalized epilepsy (IGE; including childhood absence epilepsy, juvenile absence epilepsy, juvenile myoclonic epilepsy and epilepsy with generalized tonic–clonic seizures alone) as well as other types of GGE that do not fulfill the strict criteria for IGE.^2^ The inheritance is likely complex; that is, a combination of genetic variants possibly together with environmental factors is responsible for the phenotype.^3^

Genome‐wide association studies (GWASs) identified common genetic risk variants for generalized epilepsy.^4^, ^5^ The polygenic risk score (PRS) is a measure that quantifies the impact of these genetic variants, which individually are of small effect on susceptibility, but in combination may distinguish healthy individuals from people prone to develop GGE.^6^ Although polygenic risk scores have proven useful in estimating disease susceptibility, their ability to capture clinical severity or therapeutic response in GGE remains uncertain.

Based on the dataset from the GWAS in 2018,^4^ a PRS was calculated in patients with GGE and population controls, taking 50 000–60 000 single nucleotide polymorphisms into account. Here, an enrichment of patients with epilepsy in the highest PRS burden percentile as well as a significant difference of the PRS in GGE compared to population controls was detected, indicating a predictive power of the PRS for the presence of GGE.^7^ The predictive value of PRS for GGE was confirmed in independent cohorts.^8^ Furthermore, in a population‐based study, a high PRS for GGE was significantly associated with not having a university/college degree, and a high PRS for focal epilepsy correlated with experienced depression or anxiety, indicating a pleiotropic effect of the genetic factors.^9^

Although PRS reflects the risk of developing GGE, its ability to predict specific phenotypic features (e.g., specific GGE syndrome, drug resistance) remains unknown; this gap underpins the rationale for evaluating PRS as a potential clinical prediction tool.

We hypothesized that the clinical phenotype of GGE patients with a high PRS differs from those with a low PRS.

## 2 MATERIALS AND METHODS

### 2.1 PRS calculation

The PRS used in this study are based on the PRS calculated in Leu et al.^7^ Single nucleotide polymorphism weights were derived from the GWAS summary statistics.^4^ GGE PRS of individual patients were generated using the allelic scoring function of PLINK v1.9^10^ and curtailed based on *p* ≤ .5. For Epi25 year 1–4 data, the data were filtered (quality/kinship) and selected by a match with the 1000 Genomes Project for core European. Entries in the 2018 International League Against Epilepsy (ILAE) GWAS were excluded. PRS calculations were performed using the software PRSice.

### 2.2 Study cohort

Patients with GGE were recruited by the Epi25 Collaborative project, an international, multicenter research consortium (http://epi‐25.org).^11^ From the cohort of non‐Finnish, European ancestry GGE patients (*n* = 2256) in Years 1 and 2 of the Epi25 Collaborative,^12^ 100 patients each with the highest and the lowest PRS were chosen. Contributing sites of the selected patients were approached and completed a detailed phenotyping questionnaire on age at onset, age at last follow‐up, family history, febrile seizures, pre‐/perinatal complications, epilepsy syndrome, seizure types, co‐occurrence of functional/dissociative (nonepileptic) seizures, psychiatric comorbidities, neurological examination, and electroencephalographic (EEG) and magnetic resonance imaging (MRI) findings as well as history of antiseizure medication (ASM). The data were ascertained by retrospective review of medical records by an epilepsy specialist at each site to enrich the phenotypic information available from the original Epi25 phenotypic dataset.

### 2.3 Clinical data acquisition

Detailed data were provided by 13 contributing centers for 109 of the 116 participants they had originally included into Epi25. These 109 participants consisted of 60 individuals with highest and 49 individuals with lowest PRS who were included in the study. The detailed phenotypic data provided were then reviewed. One patient in each group was excluded, as they did not fulfill the Epi25 inclusion criteria for GGE (https://epi‐25.org/epi25‐eligibility‐criteria), leading to a size of 59 individuals with highest and 48 individuals with lowest PRS.

For validation of the findings on pharmacoresistance, we matched the phenotypic data of the whole Epi25 year 1–4 GGE cohort with the GGE PRS scores of the participants with core European ancestry (*n* = 2287). European ancestry was determined based on a principal component analysis in Genome‐wide Complex Trait Analysis (GCTA) using a genetic relationship matrix constructed from linkage disequilibrium‐pruned genotype data with PLINK v1.9, as well as on projection onto principal components computed jointly with European samples from the 1000 Genomes Project.^13^, ^14^ Epilepsy syndromes and seizure semiology were classified according to the 2017 ILAE classification.^15^, ^16^ EEG was scored utilizing the revised 2017 glossary of terms.^17^

Patients who had ongoing seizures despite having tried two or more appropriately chosen and dosed ASMs were considered pharmacoresistant according to the ILAE task force definition.^18^ We extracted this information from the Epi25 dataset where contributing investigators answered yes or no to pharmacoresistant status based on the definition above. This question was answered in 51% of the GGE cases in years 1–4. Of these 1175 cases, 395 were pharmacoresistant and 780 not pharmacoresistant.

### 2.4 Statistics

Clinical data were analyzed with R statistical software (version 4.5.2). Cases of missing or not classifiable data (“unknown”) were removed from statistical analysis. Statistical analysis was performed using the Fisher exact test for count data as well as Wilcoxon rank‐sum test with continuity correction. False discovery rate (FDR) adjustment was done using the Benjamini–Hochberg procedure. Level of significance was reached at *p* < .05. To evaluate potential nonlinear associations between PRS and pharmacoresistance, we fitted a logistic regression model with a cubic regression spline (*df* = 5) and compared it with a null model using a likelihood ratio test. To address the overrepresentation of pharmacoresistant cases in our cohort compared to population‐based estimates, we performed a sensitivity analysis using bootstrap resampling (B = 1000 replicates) to approximate a pharmacoresistance prevalence of 4.5%.^19^

Association of PRS with age at afebrile seizure onset was evaluated in the Epi25 year 1–4 dataset using linear regression with PRS as continuous predictor. Model fit was compared to an intercept‐only model using a likelihood ratio test. Information on age at afebrile seizure onset was available in 1699 cases. Potential nonlinear associations were assessed by comparing the linear model to a natural cubic spline model for PRS (*df* = 5) using a likelihood ratio test.

## 3 RESULTS

We compared the clinical phenotype of GGE patients derived from the Epi25 Collaborative cohort from the spectrum of the highest PRS (*n* = 59) versus the lowest PRS (*n* = 48) of the Epi 25 year 1–2 cohorts. We did not detect significant differences in the distribution of age at onset, history of febrile seizures, pre‐/perinatal complications, epilepsy syndromes, seizure types, co‐occurrence of functional/dissociative (nonepileptic) seizures, and psychiatric comorbidities. EEG and MRI findings were also similar when comparing the subgroups (Table 1).

**TABLE 1:** Clinical phenotype of GGE patients with highest versus lowest PRS.

	Highest PRS	Lowest PRS	*p*	
			Raw	FDR‐adjusted
*n*	59	48	—	—
PRS, mean ± SD (range)	2.73 ± .59 (5.46 to 2.21)	−1.53 ± .31 SD (−1.18 to −2.54)	—	—
Age at first seizure, mean, years ± SD (range)	12.5 ± 9.1 (.7 to 52), *n* = 54	12.9 ± 4.9 (2 to 26), *n* = 42	*p* = .26^a^	*p* = .89
Age at last follow‐up, mean, years ± SD (range)	27.6 ± 16.2 (3 to 75), *n* = 57	29.2 ± 13.4 (7 to 69), *n* = 48	—	—
Family history of epilepsy [affected first degree relative]	19 (45.2% of *n* = 42)	13 (37.1% of *n* = 35)	*p* = .50	*p* = 1.00
Febrile seizures, *n*	5 (12.5% of *n* = 40)	2 (4.9% of *n* = 41)	*p* = .26	*p* = .89
Pre‐/perinatal complications, *n*	3 (9.7% of *n* = 31)	5 (17.2% of *n* = 29)	*p* = .47	*p* = 1.00
Epilepsy syndromes, *n*				
GGE [subtype not specified]	20 (34.5% of *n* = 58)	8 (16.7% of *n* = 48)	*p* = .05	*p* = .89
Epilepsy with generalized tonic–clonic seizures	4 (6.9% of *n* = 58)	7 (14.6% of *n* = 48)	*p* = .22	*p* = .89
Epilepsy with eyelid myoclonias [Jeavons syndrome]	3 (5.2% of *n* = 58)	3 (6.3% of *n* = 48)	*p* = 1.00	*p* = 1.00
Epilepsy with myoclonic absences	1 (1.7% of *n* = 58)	0 (0% of *n* = 48)	*p* = 1.00	*p* = 1.00
Febrile seizures plus/GEFS plus	1 (1.7% of *n* = 58)	1 (2.1% of *n* = 48)	*p* = 1.00	*p* = 1.00
Childhood absence epilepsy	9 (15.5% of *n* = 58)	5 (10.4% of *n* = 48)	*p* = .57	*p* = 1.00
Juvenile absence epilepsy	9 (15.5% of *n* = 58)	8 (16.7% of *n* = 48)	*p* = 1.00	*p* = 1.00
Juvenile myoclonic epilepsy	11 (19.0% of *n* = 58)	16 (33.3% of *n* = 48)	*p* = .12	*p* = .89
Seizure types, *n*				
Generalized motor	47 (79.7% of *n* = 59)	42 (87.5% of *n* = 48)	*p* = .31	*p* = .91
Generalized tonic–clonic	43 (72.9% of *n* = 59)	40 (83.3% of *n* = 48)	*p* = .25	*p* = .89
Generalized myoclonic	14 (23.7% of *n* = 59)	15 (31.3% of *n* = 48)	*p* = .39	*p* = 1.00
Generalized myoclonic–atonic	3 (5.1% of *n* = 59)	3 (6.3% of *n* = 48)	*p* = 1.00	*p* = 1.00
Generalized myoclonic–tonic–clonic	1 (1.7% of *n* = 59)	0 (0% of *n* = 48)	*p* = 1.00	*p* = 1.00
Epileptic spasms	1 (1.7% of *n* = 59)	0 (0% of *n* = 48)	*p* = 1.00	*p* = 1.00
Generalized nonmotor	33 (55.9% of *n* = 59)	29 (60.4% of *n* = 48)	*p* = .69	*p* = 1.00
Typical absence	23 (39.0% of *n* = 59)	22 (45.8% of *n* = 48)	*p* = .56	*p* = 1.00
Atypical absence	4 (6.8% of *n* = 59)	2 (4.2% of *n* = 48)	*p* = .69	*p* = 1.00
Myoclonic absence	1 (1.7% of *n* = 59)	0 (0% of *n* = 48)	*p* = 1.00	*p* = 1.00
Absence with eyelid myoclonia	5 (8.5% of *n* = 59)	3 (6.3% of *n* = 48)	*p* = .73	*p* = 1.00
Absences [not further specified]	4 (6.8% of *n* = 59)	5 (10.4% of *n* = 48)	*p* = .73	*p* = 1.00
Additional psychogenic, nonepileptic seizures, *n*	2 (3.9% of *n* = 51)	0 (.0% of *n* = 48)	*p* = .50	*p* = 1.00
Psychiatric comorbidities, *n*	8 (14.8% of *n* = 54)	12 (27.9% of *n* = 43)	*p* = .21	*p* = .89
Abnormal neurological exam, *n*	3 (6.4% of *n* = 47)	7 (15.9% of *n* = 44)	*p* = .19	*p* = .89
Routine EEG, *n*				
Nonepileptiform abnormalities	4 (17.4% of *n* = 23)	3 (7.9% of *n* = 38)	*p* = .24	*p* = .89
Background slowing	1 (4.3% of *n* = 23)	2 (5.3% of *n* = 38)	*p* = 1.00	*p* = 1.00
Continuous generalized slowing	2 (8.7% of *n* = 23)	0 (0% of *n* = 38)	*p* = .14	*p* = .89
Hypsarrhythmia	1 (4.3% of *n* = 23)	0 (0% of *n* = 38)	*p* = .38	*p* = 1.00
Regional slowing	0 (0% of *n* = 23)	1 (2.6% of *n* = 39)	*p* = 1.00	*p* = 1.00
Epileptiform abnormalities	43 (84.3% of *n* = 51)	39 (88.6% of *n* = 44)	*p* = .77	*p* = 1.00
Generalized epileptiform discharges	42 (97.7% of *n* = 43)	38 (97.4% of *n* = 39)	*p* = 1.00	*p* = 1.00
3‐Hz spike wave complexes	18 (41.9% of *n* = 43)	18 (46.2% of *n* = 39)	*p* = .82	*p* = 1.00
Polyspikes	11 (25.6% of *n* = 43)	13 (33.3% of *n* = 39)	*p* = .48	*p* = 1.00
Photoparoxysmal response	12 (35.3% of *n* = 34)	8 (19.5% of *n* = 41)	*p* = .19	*p* = .89
Hyperventilation‐induced epileptiform discharges	11 (39.3% of *n* = 28)	12 (37.5% of *n* = 32)	*p* = .31	*p* = .91
MRI of the brain, *n*				
Nonepileptogenic lesions	4 (9.3% of *n* = 43)	5 (13.2% of *n* = 38)	*p* = .72	*p* = 1.00
Epileptogenic lesions	1 (2.3% of *n* = 43)^b^	0 (0% of *n* = 38)	*p* = 1.00	*p* = 1.00
Pharmacoresistance, *n*	4 (8.9% of *n* = 45)	13 (31.7% of *n* = 41)	*p* = .01	*p* = .41

*Note*: Results are expressed as an absolute number as well as a percentage of patients whose information was available. Analysis was conducted using Fisher exact test for count data and for the analysis of the age at onset.

Abbreviations: EEG, electroencephalography; FDR, false discovery rate; GEFS, genetic epilepsy with febrile seizures; GGE, genetic generalized epilepsy; MRI, magnetic resonance imaging; PRS, polygenic risk score.

^a^ Wilcoxon rank‐sum test was used.

^b^ Mild hippocampal sclerosis, considered unrelated to the GGE.

Interestingly, more patients from the lowest compared to the highest PRS subgroup were pharmacoresistant (31.7% vs. 8.9%, *p* = .01), but this was not significant after FDR adjustment (*p* = .42; Figure 1A). In addition, a higher percentage of unspecified generalized epilepsy was seen in the high PRS cohort (*p* = .05), which did not remain significant after FDR adjustment (*p* = .91).

**FIGURE 1:**
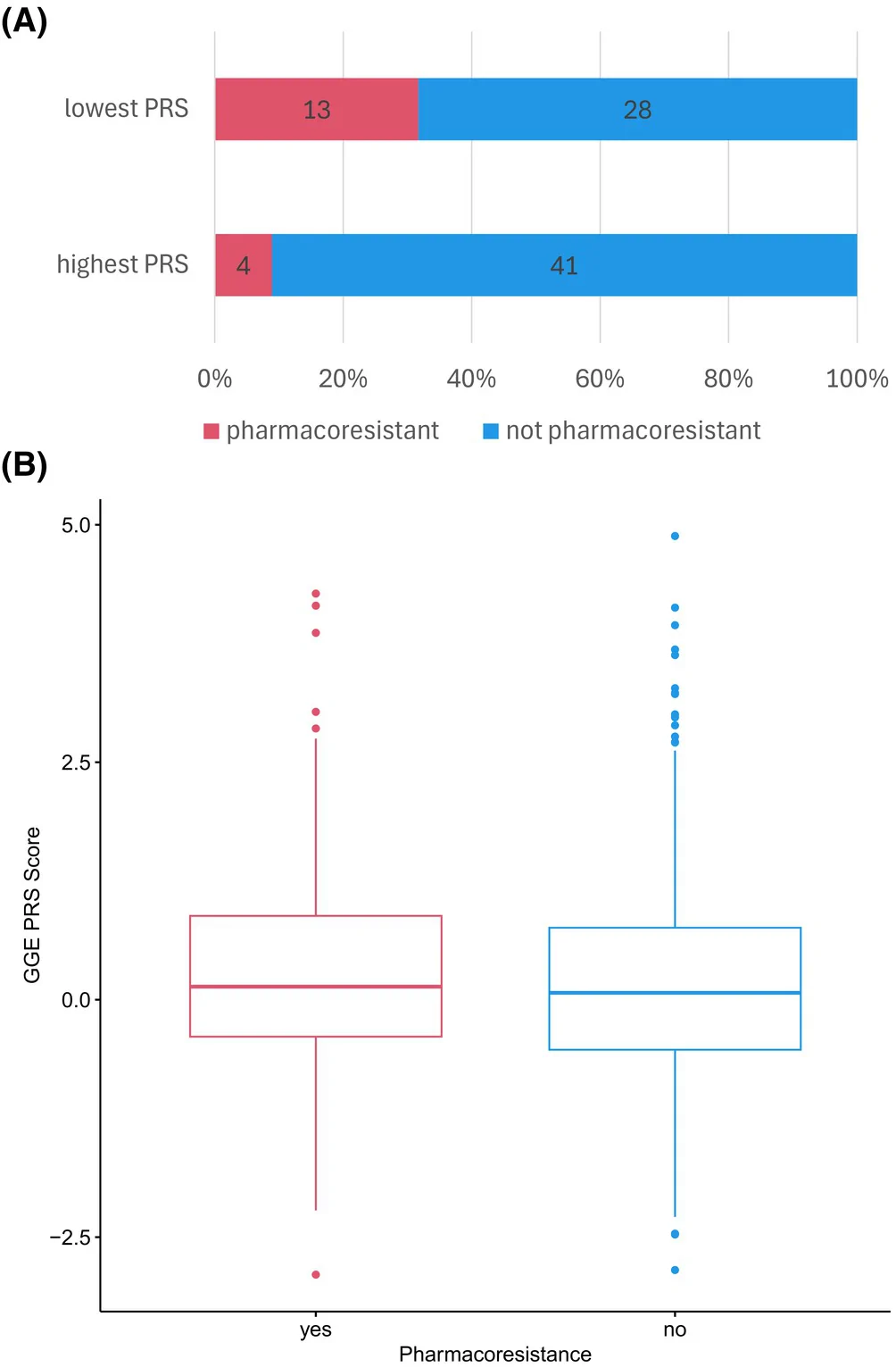
Pharmacoresponse in genetic generalized epilepsy (GGE) patients. (A) Comparison of the patients with highest and lowest polygenic risk score (PRS). Statistical analysis used Fisher exact test for count data “pharmacoresistant” versus “not pharmacoresistant” for patients with the highest (*n* = 45) versus the lowest PRS (*n* = 41), excluding “unknown” from statistical analysis. A higher number of GGE patients in the subgroup with the lowest PRS were pharmacoresistant (*p* = .01). (B) Comparison of the PRS in individuals from year 1–4 cohorts who were pharmacoresistant versus not pharmacoresistant. Epi25 phenotypic data and GGE PRS scores of the inner European cohort were matched (*n* = 1175) and divided into pharmacoresistant (*n* = 780) versus not pharmacoresistant (*n* = 395). No significant difference in the PRS was found between the subgroups using Wilcoxon rank‐sum test with continuity correction (*p* = .12).

To validate our results, we examined the link between the PRS and pharmacoresistance in a larger cohort with European ethnicity from the Epi25 Collaborative (*n* = 1175). No significant difference in PRS was detected between the pharmacoresistant (*n* = 395) versus not pharmacoresistant (*n* = 780) cohort (*p* = .12; Figure 1B). The spline model did not improve model fit compared with the null model (ΔDeviance = 3.28 with *df* = 5, *p* = .66), indicating no evidence of a nonlinear association between PRS and pharmacoresistance. The bootstrap sensitivity analysis resampling to a population‐based pharmacoresistance prevalence of 4.5%^19^ was also consistent with these results (median ΔDeviance = 6.34, 95% confidence interval [CI] = 1.23–16.88, median *p* = .27, 95% CI = .00–.94, proportion of replicates with *p* < .05: 15.8%).

PRS was not significantly associated with age at afebrile seizure onset (linear model vs. null model: *F* = 2.83, *p* = .093). There was no evidence of nonlinearity (natural cubic spline model with *df* = 5 vs. linear model: *F* = .33, *p* = .856).

## 4 DISCUSSION

This study did not show major phenotypic differences between GGE patients with the highest compared to those with the lowest PRS scores. Statistically, we initially found a tendency for a difference in favor of a better response to ASM in the cohort with the highest PRS (*p* = .01). However, these results firstly did not withstand FDR adjustment (*p* = .42) and secondly, the correlation between PRS and pharmacoresistance was not reproduced in a larger validation cohort covering the entire breadth of the PRS.

Polygenic risk can explain higher disease risk in individual patients across both common diseases^20^, ^21^ including epilepsy^22^ and overall disease burden.^23^ However, attempts to use PRS‐based disease risk stratification have not demonstrated clear results.^24^, ^25^ The overall accuracy for single traits varies considerably across ancestries and is generally low.^26^, ^27^ The link between PRS and disease severity is less well understood, although an association was previously reported for a highly correlated PRS, schizophrenia.^28^ Here, we found no meaningful association between the 2018 PRS and the severity of the GGE phenotype. Our findings are consistent with a prior genome‐wide association meta‐analysis including the Epi25 cohort, which did not identify genetic factors associated with drug‐resistant generalized epilepsy, although this study had limited power.^29^ Our results also align with another recent study that did not show a significant difference in PRS between the group of pharmacoresponders versus nonresponders and was unable to generate a PRS to estimate the likelihood of a pharmacoresponse in GGE patients.^30^ The lack of association between PRS and clinical outcomes could potentially be explained by mechanisms beyond common polygenic burden, including rare genetic variation, developmental factors, and environmental influences. However, we cannot exclude that PRS has minor effects on the investigated phenotypic features. Identification of such minor effects would require even larger cohorts.

PRS currently only explains a fraction of the heritability, and other genetic factors that are not captured using 2018 PRS are relevant as well. A recent study investigated PRS in families with a known monogenic cause for genetic epilepsy with febrile seizures plus.^31^ This study showed that family members with more severe phenotypes had higher PRS compared to their less severely affected or unaffected relatives. Hence, future improved PRS based on even larger studies that will capture most of the common variants contributing to GGE may correlate with the severity of the phenotype. Therefore, we suggest that the correlation between phenotype and PRS should be examined again in the future using improved PRS from future GWASs.

The strength of our study is the detailed phenotyping of a selection of patients from the two extremes of the PRS spectrum. This was done to increase power to detect phenotypic differences, as these were expected to be most pronounced at the extremes of the PRS spectrum. However, even using this strategy, our hypothesis that patients with a high PRS would have certain, probably more severe, phenotypes was not confirmed. However, our study may have been underpowered and unable to recognize minor phenotypic differences. Another limitation of our study is that the PRS scores used were based on the 2018 GWAS.^4^ The most recent GWAS from 2023^5^ included the Epi25 cohort and could therefore not be used to develop the PRS for this study. We would also like to point out that we used Epi25 inclusion criteria for GGE. These align well with the current ILAE criteria for GGE^2^ but are broader than criteria for IGE that were published after the inception of Epi25.^2^ Hence, our findings may not be representative for a pure IGE cohort. Furthermore, the Epi25 participants were recruited from tertiary epilepsy centers, likely resulting in a selection of overall more severe cases with higher percentage of pharmacoresistance. However, a bootstrap sensitivity analysis to approximate the population‐based pharmacoresistance prevalence of 4.5% reported for GGE patients^19^ confirmed the absence of a nonlinear association between PRS and pharmacoresistance, suggesting that our findings are robust to this referral bias.

## 5 CONCLUSIONS

Although two cohorts of individuals with the highest versus the lowest PRS were compared, the level of the PRS did not show significant correlation with the phenotype of GGE patients. Although an increased GGE PRS is conceptualized to predict a higher risk of epilepsy, no meaningful association with the severity of the phenotype could be demonstrated in this study. At present, PRSs should be regarded as a research tool rather than a clinically actionable biomarker for prognostic stratification or treatment decision‐making in GGE. Future work may leverage machine learning models that integrate PRS with clinical, EEG, and imaging data to explore multidimensional risk stratification.

## AUTHOR CONTRIBUTIONS

Sophie von Brauchitsch, Nils Hartung, Felix Rosenow, and Karl Martin Klein are responsible guarantors for the overall article and contributed to the conception and design of the study; to the acquisition, analysis, and interpretation of data; and to drafting the manuscript and figures. Samuel F. Berkovic, Bobby Koeleman, Dennis Lal, and Costin Leu contributed to the conception and design of the study, contributed and revised the manuscript, and gave final approval of the article. Stefan Wolking and Robin A. Karge provided processed data and contributed to the analysis. Robin A. Karge, Ingrid E. Scheffer, Danielle M. Andrade, Savvas S. Papacostas, Wolfram S. Kunz, Ingo Helbig, Hiltrud Muhle, Christian Boßelmann, Yvonne Weber, Holger Lerche, Mark I. Rees, Sanjay M. Sisodiya, Pasquale Striano, Francesca Bisulli, Antonio Gambardella, Renzo Guerrini, Lynnette Sadleir, Philipp S. Reif, and Stefan Wolking contributed to the acquisition and analysis of the data and approved the final version. All authors have reviewed and approved the content of the manuscript. Requirements for authorship are met by all authors.

## FUNDING INFORMATION

F.R., K.M.K., and S.v.B. declare financing through the LOEWE Program of the State of Hesse, Germany, and the Detlev‐Wrobel‐Fonds for Epilepsy Research Frankfurt, Germany. No specific grants for this study from the commercial sector have been received. Epi25 is part of the Centers for Common Disease Genomics (CCDG) program, funded by the National Human Genome Research Institute (NHGRI) and the National Heart, Lung, and Blood Institute. CCDG‐funded Epi25 research activities at the Broad Institute, including genomic data generation in the Broad Genomics Platform, are supported by NHGRI grant UM1 HG008895 (principal investigators: Eric Lander, Stacey Gabriel, Mark Daly, Sekar Kathiresan). The Genome Sequencing Program efforts were also supported by NHGRI grant 5U01HG009088–02. S.W. and R.A.K. received funding from the German Research Foundation (WO 2385/2–1). L.S. was supported by the Health Research Council of New Zealand and Cure Kids. Y.W. received funding from the German Research Ministry (BMBF TreatIon 01GM2210B) and the German Research Foundation (WE 4896/4–2). M.I.R. was funded by Health & Care Research Wales (Wales Epilepsy Research Network) and Wales Gene Park. I.H. received funding from the German Research Foundation (HE 5415/7–2).

## CONFLICT OF INTEREST STATEMENT

F.R. reports personal fees from Angelini Pharma, Desitin Pharma, Eisai, Jazz Pharma, Roche Pharma, Stoke Therapeutics, and UCB Pharma and grants from the Detlev‐Wrobel Fonds for Epilepsy Research, the Deutsche Forschungsgemeinschaft, the Federal Ministry of Education and Research, the LOEWE Program of the State of Hesse, and the European Union. K.M.K. reports personal fees from UCB Pharma and Jazz Pharmaceuticals. L.S. reports consulting fees from the Epilepsy Study Consortium. I.H. has received funding from the German Research Foundation (HE 5415/7–2). None of the other authors has any conflict of interest to disclose.

## ETHICS STATEMENT

Ethics approval was obtained from the ethics committee of the Medical Faculty of the Goethe University Frankfurt (ID#19–523) and the local institutional review boards of all contributing centers. This study was performed in accordance with the Declaration of Helsinki. We confirm that we have read the Journal's position on issues involved in ethical publication and affirm that this report is consistent with those guidelines.

## PATIENT CONSENT STATEMENT

All participants provided written informed consent.

## Supporting information


Appendix S1.

## Data Availability

The data that support the findings of this study are openly available in NHGRI's controlled‐access AnVIL platform at https://anvilproject.org/.
