# Supplementary material for: Correlation of polygenic risk score and clinical phenotype in patients with genetic generalized epilepsy
Source: Epilepsia. 2026 Aug 22;67(11):6005–14. doi: 10.1002/epi.70444 (PMC13768941; doi:10.1002/epi.70444)
Supplement: Supplementary file 1 [file EPI-67-6005-s001.docx]

**Appendix – Contributing sites**

Karl Martin Klein

Departments of Clinical Neurosciences, Medical Genetics and Community Health Sciences Hotchkiss Brain Institute & Alberta Children's Hospital Research Institute, Cumming School of Medicine, University of Calgary, Calgary, Canada

Costin Leu, Dennis Lal

Genomic Medicine Institute, Lerner Research Institute Cleveland Clinic, Cleveland (Ohio), USA

Costin Leu

Department of Clinical and Experimental Epilepsy, Institute for Neurology, University College London, London, UK

Costin Leu, Dennis Lal

Stanley Center for Psychiatric Research, Broad Institute of Harvard and M.I.T, Cambridge (Massachusetts), USA

Dennis Lal

Cologne Center of Genomics, University of Cologne, Cologne Germany

Department of Neurology, McGovern Medical School, The University of Texas Health Science

Center at Houston, Houston, Texas, USA

Bobby Koeleman

Department of Medical Genetics, University Medical Center Utrecht, Utrecht University, Utrecht Netherlands

Sam Berkovic, Ingrid Scheffer

Epilepsy Research Centre, Melbourne Brain Centre (Austin Health), University of Melbourne, Melbourne, Australia

Ingrid Scheffer

Department of Neurology, Royal Children's Hospital, Melbourne Australia

Florey Institute of Neuroscience and Mental Health, University of Melbourne, Melbourne Australia

Danielle Andrade, Quratulain Zulfiqar Ali

Epilepsy Clinic, Krembil Brain Institute, University Health Network, University of Toronto, Toronto, Canada

Savvas S Papacostas, Ioanna Kousiappa, Andreas M Koupparis

Neurophysiology Department, Cyprus Institute of Neurology & Genetics, Nicosia, Cyprus

George A Tanteles

Clinical Genetics and Genomics Department, Cyprus Institute of Neurology & Genetics, Nicosia, Cyprus

Department of Basic and Clinical Sciences, University of Nicosia Medical School, University of Nicosia, Nicosia, Cyprus

Wolfram S. Kunz

Department of Epileptology, University Bonn Medical Center, University Bonn, Bonn Germany

Ingo Helbig, Hiltrud Muhle, Manuela Pendziwiat

Department of Neuropediatrics, University Medical Center Schleswig-Holstein, Christian-Albrecht University of Kiel, Kiel, Germany

Ingo Helbig

Division of Neurology, Children's Hospital of Philadelphia, Philadelphia (Pennsylvania), USA

Epilepsy NeuroGenetics Initiative, Children's Hospital of Philadelphia Research Institute, Philadelphia (Pennsylvania) USA

Department of Neurology, Institute for Translational Medicine and Therapeutics, Perelman School of Medicine, University of Pennsylvania, Philadelphia (Pennsylvania), USA

Penn Presbyterian Medical Center, University of Pennsylvania, Philadelphia (Pennsylvania), USA

Holger Lerche, Christian Hengsbach, Christian M Boßelmann

Department of Neurology and Epileptology, Hertie Institute for Clinical Brain Research, University Clinic Tübingen, Eberhard Karl University of Tübingen, Tübingen, Germany

Stefan Wolking, Yvonne Weber

Section of Epileptology, Department of Neurology, RWTH Aachen University, Aachen, Germany

Mark I. Rees

Neurology Research Group, Institute of Life Science, Faculty of Medicine, Health & Life Sciences, Swansea University, South Wales, UK

Brain and Mind Centre, Faculty of Medicine and Health, University of Sydney, Sydney, Australia

Sanjay M Sisodiya

Research Department of Epilepsy, UCL Queen Square Institute of Neurology, University College London, London, UK

Chalfont Centre for Epilepsy, Chalfont St Peter, UK

Pasquale Striano, Federico Zara

IRCCS Istituto G. Gaslini, full member of ERN-EPICARE, Genova Italy Department of Neuroscience, Rehabilitation, Ophthalmology, Genetics and Mother-Child-Sciences DINOGMI, University of Genova, Genova, Italy

Francesca Bisulli

IRCCS Istituto delle Scienze Neurologiche di Bologna (Full Member of the ERN EpiCARE Bologna Italy

Department of Biomedical and Neuromotor Sciences, University of Bologna, Bologna, Italy

Antonio Gambardella

Department of Medical and Surgical Sciences, Magna Græcia University of Catanzaro, Catanzaro, Italy

Renzo Guerrini

Pediatric Neurology Unit, A. Meyer Children's Hospital, University of Florence, Florence, Italy

Lynette Sadleir

Department of Paediatrics and Child Health, University of Otago, Wellington, New Zealand

**Appendix – Epi25 Collaborative**

Siwei Chen^1^, Quratulain Zulfiqar Ali^2^, Elisabetta Amadori^3^, Alison Anderson^4, 5^, Danielle M. Andrade^2^, Mutluay Arslan^6^, Mark D. Baker^7^, Ganna Balagura^8^, Simona Balestrini^9, 10^, Carmen Barba^11^, Larry W. Baum^12^, Tobias Baumgartner^13^, Betül Baykan^14^, Felicitas Becker^15, 16^, Caitlin A. Bennett^17^, Ahmad Beydoun^18^, Claudia Bianchini^11^, Francesca Bisulli^19, 20^, Ingo Borggraefe^21^, Christian Bosselmann^15^, Knut Brockmann^22^, Robyn M. Busch^23-25^, Laura Canafoglia^26^, Barbara Castellotti^27^, Andrew J. Cole^28^, Duccio Cordelli^20, 29^Patrick Cossette^30^, Chantal Depondt^31^, Orrin Devinsky^32^, Lidia Di Vito^20^, Dennis J. Dlugos^33, 34^, Viola Doccini^11^, Hany El-Naggar^35, 36^, Colin A. Ellis^37^, Thomas N. Ferraro^38, 39^, Lorenzo Ferri^20^, Francesco Fortunato^40^, Jacqueline A. French^32^, Elena Freri^41^, Antonio Gambardella^40^, Tania Giangregorio^42^, Alica Goldman^43^, Tiziana Granata^41^, Renzo Guerrini^11, 44^, Aslı Gundogdu-Eken^45^, Namrata Gupta^46^, Kevin Haas^47^, Manu Hegde^48^, Erin L. Heinzen^49^, Ingo Helbig^33, 37, 50-53^, Christian Hengsbach^15^, Henrike Heyne^46, 54^, Olivia Hoeper^17^, Reetta Kälviäinen^55, 56^, Yeşim Kesim^14^, Karl Martin Klein^57-63^, Andreas Koupparis^64^, Ioanna Kousiappa^64^, Roland Krause^65^, Martin Krenn^66^, Wolfram S. Kunz^13, 67^, Patrick Kwan^4, 5, 68^, Austin Lacey^35, 69^, Dennis Lal^23, 24^, Stephanie L. Leech^17^, Holger Lerche^15^, Costin Leu^9, 23^, Calwing Liao^46, 70-72^, Laura Licchetta^20, 29^, Kuang-Lin Lin^73^, Tarja Linnankivi^74-76^, Warren Lo^77^, Daniel H. Lowenstein^48^, Francesca Madia^3^, Stefania Magri^78^, Louis Maillard^79^, Ida Manna^80^, Paula Marques^81^, Anthony G. Marson^82^, Raffaella Minardi^20^, Barbara Mostacci^20^, Lorenzo Muccioli^20^, Hiltrud Muhle^50^, Wassim Nasreddine^18^, Terence J. O'Brien^4, 5^, Çiğdem Özkara^83^, Savvas S. Papacostas^64^Manuela Pendziwiat^50, 51^, Tommaso Pippucci^42^, Annapurna Poduri^84^, Robert H.W. Powell^7, 85^, Michael Privitera^86^, Mark I. Rees^7, 87^, Philipp S. Reif^62, 63^, Antonella Riva^3, 8^, Felix Rosenow^62, 63, 88^, Philippe Ryvlin^89^, Lynette G. Sadleir^90^, Martina Soldà^20^, Andrea Salmon^57^, Ilaria Sammarra^91^, Marcello Scala^3, 8^, Steven Schachter^92^, Ingrid E. Scheffer^17, 93, 94^, Susanne Schubert-Bast^62, 63, 95^, Paolo Scudieri^3, 8^, Graeme J. Sills^96^, Sanjay M. Sisodiya^9, 10^, Bernhard J. Steinhoff^97^, Ulrich Stephani^50^, Carlotta Stipa^20^, Pasquale Striano^3, 8^, Adam Strzelczyk^62^, Rainer Surges^13^, Toshimitsu Suzuki^98, 99^, Mariagrazia Talarico^91^, George A. Tanteles^100, 101^, Marian Todaro^4, 5^, Pınar Topaloğlu^102^, Meng-Han Tsai^103^, Birute Tumiene^104, 105^, Dilsad Turkdogan^106^, Luc Valton^107^, Andreas van Baalen^50^, Annalisa Vetro^11^, Yvonne G. Weber^15, 108^, Sarah Weckhuysen^109-111^, Peter Widdess-Walsh^35, 36, 69^, Samuel Wiebe^57, 58, 60, 112, 113^, Stefan Wolking^15, 108^, Randi von Wrede^13^, Kazuhiro Yamakawa^98, 99^, Zuhal Yapıcı^102^, Felix Zahnert^63, 88^, Fritz Zimprich^66^, Milena Zizovic^65^, Gábor Zsurka^13, 67^, Benjamin M. Neale^46, 70, 71^, Samuel F. Berkovic^17^

1.	Department of Human Genetics, The University of Chicago, Chicago, Illinois, USA.

2.	Adult Genetic Epilepsy Program, Division of Neurology, Krembil Brain Institute, University University of Toronto, Toronto Canada.

3.	IRCCS Istituto Giannina Gaslini, Genova, Italy.

4.	Department of Medicine, University of Melbourne, Royal Melbourne Hospital, Parkville 3050, Australia.

5.	Department of Neuroscience, The School of Translational Medicine, Alfred Health, Monash University, Melbourne 3004, Australia.

6.	Department of Pediatric Neurology, Gülhane Training and Research Hospital of the University of Health Sciences, Ankara, 06010, Turkey.

7.	Swansea University Medical School, Swansea University, Swansea, Wales, UK.

8.	Department of Neurosciences, Rehabilitation, Ophthalmology, Genetics, Maternal and Child Health, University of Genova, Genova, Italy.

9.	Department of Clinical and Experimental Epilepsy, UCL Queen Square Institute of Neurology, London WC1N 3BG, UK.

10.	Chalfont Centre for Epilepsy, Chalfont-St-Peter, Buckinghamshire SL9 0RJ, UK. .

11.	Neuroscience Department, Meyer Children’s Hospital IRCCS, Florence, Italy.

12.	Department of Psychiatry, The University of Hong Kong, Pokulam, Hong Kong.

13.	Department of Epileptology, University of Bonn Medical Centre, Bonn 53127, Germany.

14.	Department of Neurology, Istanbul Faculty of Medicine, Istanbul University, Istanbul, Turkey.

15.	Department of Neurology and Epileptology, Hertie Institute for Clinical Brain Research, University of Tübingen, Tübingen 72076, Germany.

16.	Department of Neurology, University of Ulm, Ulm 89081, Germany.

17.	Epilepsy Research Centre, University of Melbourne, Austin Health, Heidelberg 3084, Australia.

18.	Department of Neurology, American University of Beirut Medical Center, Beirut, Lebanon.

19.	Department of Biomedical and Neuromotor Sciences, University of Bologna, Bologna, Italy.

20.	IRCCS Istituto delle Scienze Neurologiche di Bologna, (Reference Center for Rare and Complex Epilepsies - EpiCARE), Bologna, Italy.

21.	Department of Pediatric Neurology, Dr von Hauner Children's Hospital, Ludwig Maximilians University, Munchen, Germany.

22.	Children's Hospital, Dept. of Pediatric Neurology, University Medical Center Göttingen, Göttingen, Germany.

23.	Genomic Medicine Institute, Lerner Research Institute, Cleveland Clinic, Cleveland, OH 44195, USA.

24.	Cleveland Clinic Epilepsy Center, Neurological Institute, Cleveland Clinic, Cleveland, OH 44195, USA.

25.	Department of Neurology, Neurological Institute, Cleveland Clinic, Cleveland, OH 44195, USA.

26.	Integrated Diagnostics for Epilepsy, Fondazione IRCCS Istituto Neurologico C. Besta, Milan, Italy.

27.	Unit of Medical Genetics and Neurogenetics, Department of Diagnostic and Technology, Fondazione IRCCS Istituto Neurologico Carlo Besta Milano, Italy.

28.	Neurology, Massachusetts General Hospital, Boston, MA, USA.

29.	Department of Medical and Surgical Sciences, University of Bologna, Bologna, Italy.

30.	Department of Neurosciences, Université de Montréal, Montréal, CA 26758, Canada.

31.	Department of Neurology, CUB Erasme Hospital, Hôpital Universitaire de Bruxelles (H.U.B.), Université Libre de Bruxelles (ULB), 1070 Brussels, Belgium.

32.	Department of Neurology, New York University/Langone Health, New York NY, USA.

33.	Division of Neurology, Children’s Hospital of Philadelphia, Philadelphia, 3401 Civic Center Blvd, Philadelphia, PA 19104, USA.

34.	Perelman School of Medicine, University of Pennsylvania, Philadelphia, PA, USA.

35.	The FutureNeuro Research Centre, Dublin, Ireland.

36.	Department of Neurology, Beaumont Hospital, Dublin D09 FT51, Ireland.

37.	Department of Neurology, University of Pennsylvania, Perelman School of Medicine, Philadelphia, PA, 19104 USA.

38.	Department of Biomedical Sciences, Cooper Medical School of Rowan University Camden, NJ 08103, USA.

39.	Department of Pharmacology and Psychiatry, University of Pennsylvania Perlman School of Medicine, Philadelphia, PA 19104, USA.

40.	Institute of Neurology, Department of Medical and Surgical Sciences, University “Magna Graecia”, Catanzaro, Italy.

41.	Department of Pediatric Neuroscience, Fondazione IRCCS Istituto Neurologico Carlo Besta, Milan, Italy.

42.	IRCCS Azienda Ospedaliero-Universitaria di Bologna, Medical Genetics Unit, Bologna, Italy.

43.	Department of Neurology, Baylor College of Medicine.

44.	Department of NEUROFARBA, University of Florence, Florence, Italy.

45.	Department of Molecular Biology and Genetics, Bogaziçi University, Istanbul, Turkey.

46.	Program in Medical and Population Genetics, Broad Institute of MIT and Harvard, Cambridge, MA, USA.

47.	Department of Neurology, Vanderbilt University Medical Center, Nashville, TN, USA.

48.	Department of Neurology, University of California, San Francisco, CA 94143, USA.

49.	Division of Pharmacotherapy and Experimental Therapeutics, Eshelman School of Pharmacy, University of North Carolina at Chapel Hill, Chapel Hill, NC, 27599, USA.

50.	Department of Neuropediatrics, University Medical Center Schleswig-Holstein, Christian-Albrechts-University, Kiel, Germany.

51.	Institute of Clinical Molecular Biology, Christian-Albrechts-University of Kiel, Kiel, Germany.

52.	The Epilepsy NeuroGenetics Initiative (ENGIN), Children's Hospital of Philadelphia, Philadelphia, 3401 Civic Center Blvd, Philadelphia, PA 19104, USA.

53.	Department of Biomedical and Health Informatics (DBHi), Children’s Hospital of Philadelphia, Philadelphia, PA, 19104 USA.

54.	Hasso Plattner Institute, Digital Engineering Faculty, University of Potsdam, Germany.

55.	Kuopio Epilepsy Center, Neurocenter, Kuopio University Hospital, Kuopio 70210, Finland.

56.	Institute of Clinical Medicine, University of Eastern Finland, Kuopio 70210, Finland.

57.	Department of Clinical Neurosciences, Cumming School of Medicine, University of Calgary, Calgary, Alberta, Canada.

58.	Department of Community Health Sciences, Cumming School of Medicine, University of Calgary, Calgary, Alberta, Canada.

59.	Department of Medical Genetics, Cumming School of Medicine, University of Calgary, Calgary, Alberta, Canada.

60.	Hotchkiss Brain Institute, Cumming School of Medicine, University of Calgary, Calgary, Alberta, Canada.

61.	Alberta Children's Hospital Research Institute, University of Calgary, Calgary, Alberta, Canada.

62.	Epilepsy Center Frankfurt Rhine-Main, Center of Neurology and Neurosurgery, Goethe University Frankfurt, Frankfurt, Germany.

63.	LOEWE Center for Personalized Translational Epilepsy Research (CePTER), Goethe University Frankfurt, Germany.

64.	Neurophysiology Department, Cyprus Institute of Neurology & Genetics, Nicosia, Cyprus.

65.	Luxembourg Centre for Systems Biomedicine, University of Luxembourg, Esch-sur-Alzette L-4362, Luxembourg.

66.	Department of Neurology, Medical University of Vienna, Vienna 1090, Austria.

67.	Institute of Experimental Epileptology and Cognition Research, Medical Faculty, University of Bonn, Bonn, Germany.

68.	Department of Medicine and Therapeutics, Chinese University of Hong Kong, Hong Kong, China.

69.	School of Pharmacy and Biomolecular Sciences, The Royal College of Surgeons in Ireland, Dublin, Ireland.

70.	Analytic and Translational Genetics Unit, Department of Medicine, Massachusetts General Hospital and Harvard Medical School, Boston, MA 02114, USA.

71.	Stanley Center for Psychiatric Research, Broad Institute of MIT and Harvard, Cambridge, MA, USA.

72.	Center for Genomic Medicine, Massachusetts General Hospital, Boston, MA, USA.

73.	Department of Pediatric Neurology, Chang Gung Memorial Hospital, Taoyuan, Taiwan.

74.	Child Neurology, New Children´s Hospital, Helsinki, Finland.

75.	Pediatric Research Center, University of Helsinki, Helsinki, Finland.

76.	Helsinki University Hospital, Helsinki, Finland.

77.	Department of Pediatrics and Neurology, Nationwide Children's Hospital, Columbus, OH, USA.

78.	Unit of Genetics of Neurodegenerative and Metabolic Diseases, Fondazione IRCCS Istituto Neurologico Carlo Besta, Milan, Italy.

79.	Neurology Department, University Hospital of Nancy, UMR 7039, CNRS, Lorraine University, Nancy, France.

80.	Institute of Molecular Bioimaging and Physiology, CNR, Section of Germaneto, Catanzaro, Italy.

81.	University Health Network, University of Toronto, Toronto, ON, Canada.

82.	Department of Pharmacology and Therapeutics, University of Liverpool, Liverpool L69 3GL, UK.

83.	Istanbul University-Cerrahpaşa, Cerrahpaşa Medical Faculty, Department of Neurology, Istanbul, Turkey.

84.	Epilepsy Genetics Program, Division of Epilepsy and Clinical Neurophysiology, Department of Neurology, Boston Children's Hospital, Boston, MA, USA.

85.	Department of Neurology, Morriston Hospital, Swansea Bay University Bay Health Board, Swansea, Wales, UK.

86.	Department of Neurology, Gardner Neuroscience Institute, University of Cincinnati Medical Center, Cincinnati, OH 45220, USA.

87.	Brain & Mind Centre, Faculty of Medicine & Health, University of Sydney, Sydney, New South Wales, Australia.

88.	Epilepsy Center Hessen-Marburg, Department of Neurology, Philipps University Marburg, Marburg, Germany.

89.	Department of Clinical Neurosciences, Centre Hospitalo-Universitaire Vaudois, Lausanne, Switzerland.

90.	Department of Paediatrics and Child Health, University of Otago, Wellington, New Zealand.

91.	Department of Medical and Surgical Sciences, Neuroscience Research Center, Magna Graecia University,

Catanzaro, Italy.

92.	Departments of Neurology, Beth Israel Deaconess Medical Center, Massachusetts General Hospital, and Harvard Medical School, Boston, MA 02215, USA. .

93.	Florey and Murdoch Children’s Research Institutes, Parkville, Victoria 3052, Australia.

94.	Department of Paediatrics, The University of Melbourne, Royal Children’s Hospital, Parkville, Victoria 3052, Australia.

95.	Department of Neuropediatrics, Children’s Hospital, Goethe University Frankfurt, Frankfurt, Germany.

96.	School of Life Sciences, University of Glasgow, Glasgow G12 8QQ, UK.

97.	Epilepsy Center Kork, Kehl-Kork 77694, Germany.

98.	Department of Neurodevelopmental Disorder Genetics, Institute of Brain Science, Nagoya City University Graduate School of Medical Science, Nagoya, Aichi, Japan.

99.	Laboratory for Neurogenetics, RIKEN Center for Brain Science, Wako, Saitama, Japan.

100.	Clinical Genetics and Genomics Department, Cyprus Institute of Neurology & Genetics, Nicosia, Cyprus.

101.	Department of Basic and Clinical Sciences, University of Nicosia Medical School, University of Nicosia, Nicosia, Cyprus.

102.	Department of Child Neurology, Istanbul Faculty of Medicine, Istanbul University, Istanbul, Turkey.

103.	Department of Neurology, Kaohsiung Chang Gung Memorial Hospital, Kaohsiung, Taiwan.

104.	Centre for Medical Genetics, Vilnius University Hospital Santaros Klinikos, Vilnius, Lithuania.

105.	Institute of Biomedical Sciences, Faculty of Medicine, Vilnius University, Vilnius, Lithuania.

106.	Department of Child Neurology, Medical School, Marmara University, Istanbul, Turkey.

107.	Department of Neurology, Toulouse University Hospital, Brain and Cognition Research Center (CerCo), Centre National de la Recherche Scientifique (CNRS), UMR5549, Toulouse, France.

108.	Department of Neurology and Epileptology, University of Aachen, Aachen 52074, Germany.

109.	Applied & Translational Neurogenomics Group, VIB Center for Molecular Neurology, VIB, Antwerp, Belgium.

110.	Department of Neurology, Antwerp University Hospital, Edegem 2650, Belgium.

111.	Translational Neurosciences, Faculty of Medicine and Health Science, University of Antwerp, Antwerp, Belgium.

112.	O'Brien Institute for Public Health, University of Calgary, Calgary, Alberta, Canada.

113.	Clinical Research Unit, Cumming School of Medicine, University of Calgary, Calgary, Alberta, Canada.
